# Proteome data of whole saliva which are associated with development of oral mucositis in head and neck cancer patients undergoing radiotherapy

**DOI:** 10.1016/j.dib.2016.05.053

**Published:** 2016-05-30

**Authors:** Nico Jehmlich, Petra Stegmaier, Claas Golatowski, Manuela Gesell Salazar, Christian Rischke, Michael Henke, Uwe Völker

**Affiliations:** a Department of Functional Genomics, Interfaculty Institute for Genetics and Functional Genomics, University Medicine Greifswald, Germany; b Department of Radiation Oncology, University Medical Center Freiburg, Germany

**Keywords:** Prospective study, Oral mucositis, Radiotherapy, Radiochemotherapy, Whole saliva, Head and neck cancer

## Abstract

Saliva as major human body fluid may act as an indicator of oral disease status. Oral mucositis is a common and often treatment-limiting side effect of radiotherapy for head and neck cancer patients. In this dataset, we provide the complete proteome dataset (raw and search files) of the patients at baseline of radiotherapy treatment in patients undergoing radiotherapy analyzed by nano liquid chromatography coupled to mass spectrometry (LC–MS/MS). In the data set, 5323 tryptic peptides were identified which can be assigned to 487 distinct proteins (≥2 peptides). The MS data have been deposited to the ProteomeXchange (“*ProteomeXchange provides globally coordinated proteomics data submission and dissemination*” [1]) via the PRIDE partner repository with the dataset identifier PRIDE: PXD003230. The data are associated with the previously published work, “*Differences in the whole saliva baseline proteome profile associated with development of oral mucositis in head and neck cancer patients undergoing radiotherapy*” [2].

**Specifications Table**

**Table:** 

Subject area	*Biology*
More specific subject area	*Clinical Proteomics*
Type of data	*Table*
How data was acquired	*Data were recorded using nano-liquid chromatography coupled to an LTQ Orbitrap Velos mass spectrometer*
Data format	1) *raw (Thermo instrument output files)* 2) *.msf (Proteome Discoverer output files)*
Experimental factors	*Patients received Intensity-modulated radiation (IMRT). Whole saliva was collected from patients at least three days before the radiotherapy started.*
Experimental features	1) *Whole saliva collection* 2) *Protein precipitation* 3) *Proteolytic digestion using trypsin* 4) *LC–MS/MS analysis*
Data source location	*Greifswald, Mecklenburg – West Pomerania, Germany*
Data accessibility	*Data are within this article and deposited to the ProteomeXchange Consortium via the PRIDE partner repository with the dataset identifier PRIDE: PXD003230.*

**Value of the data**

• Protein signatures of whole saliva from head and neck cancer patients are useful to differentiate between healthy and disease subjects screening for signatures of radiation associated oral mucositis (OM).
• Proteomics revealed correlation of the abundance of 48 of the 488 proteins identified with the risk of developing OM which allow further investigations towards biomarker assays.
• Whole saliva protein profiles at baseline of radiotherapy might allow identification of patients prone to adverse reactions.

## 1 Data

In this dataset, we measured the whole saliva proteome of patients for differences related to the development of oral mucositis. The data present here are (i) LC–MS/MS raw data and (ii) database search files of 50 head and neck cancer patients.

## 2 Experimental design, materials and methods

We collected unstimulated whole saliva of 50 patients before receiving radiotherapy. After treatment, 41 out of 50 patients developed oral mucositis (grade III) during radiotherapy, of which 14 patients even displayed an early oral mucositis (grade III) at low radiation dose of 30 Gy. Nine patients did not develop OM (grade III). The whole set were prepared for LC–MS/MS analysis and the acquired data are provided.

## 3 Sample preparation for proteome characterization

The volume of whole saliva ranged from 0.2 to 1.7 mL (average 0.9±0.3 mL). To acquire this proteome dataset, 0.5 mL aliquots of whole saliva proteins were precipitated using trichloroacetic acid (TCA) at a final concentration of 10% (v/v) and dithiothreitol (0.12% w/v) as described [3]. Briefly, protein pellets were resuspended in 8 M urea/ 2 M thiourea buffer. For MS-analysis, 4 µg of protein lysate were reduced (2.5 mM DTT for 1 h at 60 °C) and alkylated (10 mM iodacetamide for 30 min at 37 °C). Proteolysis was performed overnight using trypsin (Promega, Madison, WI, USA). Tryptic cleavage was stopped by adding 1% acetic acid followed by desalting and purification using ZipTip-µC18 tips (Millipore, Billerica, MA, USA).

## 4 Mass spectrometric analysis

The 50 patient samples were measured in a randomized design. Proteolytically cleaved peptides were separated prior to MS analyses by reverse phase nano HPLC on a 15 cm Acclaim PepMap100-column (C18, 3 µm, 100 Å) using an EASY-nLC Proxeon system (Thermo Scientific, Waltham, MA, USA) at a constant flow rate of 300 nL/min. The LC separation was achieved using a linear gradient of buffer B from 5% up to 25% within 63 min with 0.1% acetic acid, 2% acetonitrile in water (solvent A) and 0.1% acetic acid in 100% acetonitrile (solvent B). Peptides were measured using an LTQ Orbitrap Velos instrument (Thermo Scientific) equipped with a nano electrospray ion source operated with PicoTip Emitters (New Objective, Woburn, MA, USA). After a first survey scan with a resolution of 30,000, the MS/MS data were measured for the top 20 mass peaks in the linear ion trap at collision induced energy (CID) of 35%. The raw MS-data were processed using the Refiner MS v7.5 module (Genedata, Basel, Switzerland). Peak lists were searched against a human FASTA-formatted database containing 20,268 unique entries (human_uniprot_swissprot_2011_10.fasta) using an in-house Mascot server v2.3.2 (Matrix Science, London, GB). Database searches were performed with carbamidomethyl on cysteine as fixed modification and oxidation on methionine as variable modification. Enzyme specificity was selected to trypsin with up to two missed cleavages allowed using 10 ppm peptide ion tolerance and 0.6 Da MS/MS tolerances. Only ranked 1 peptide hits and a Mascot ion score >23 were considered as identified (Table 1).

## Figures and Tables

**Table 1:** Proteome data that are provided at PRIDE.

**Sample number**	**LC–MS/MS file available**	**Database search file available**	**Localization**	**Tumor stage**	**Oral mucositis developed**
1	x	x	Oropharynx	IVa	yes
2	x	x	Oropharynx	IVa	no
3	x	x	Hypopharynx	IVa	yes
4	x	x	Hypopharynx	IVa	yes
5	x	x	Larynx	IVb	yes
6	x	x	Hypopharynx	IVa	yes
7	x	x	Oropharynx	III	yes
8	x	x	Oropharynx	IVa	yes
9	x	x	Oropharynx	IVa	yes
11	x	x	Oropharynx	IVb	yes
12	x	x	Hypopharynx	IVa	yes
13	x	x	Oropharynx	IVa	no
14	x	x	Larynx	0	no
15	x	x	Larynx	IVa	yes
16	x	x	Hypopharynx	IVa	no
17	x	x	Larynx	IVb	yes
18	x	x	Hypopharynx	IVa	yes
19	x	x	Hypopharynx	IVa	no
20	x	x	Oropharynx	IVa	yes
21	x	x	Oropharynx	IVa	yes
22	x	x	Oropharynx	IVa	yes
23	x	x	Oropharynx	IVa	yes
24	x	x	Oropharynx	IVa	yes
25	x	x	Larynx	IVa	yes
26	x	x	Hypopharynx	IVa	yes
27	x	x	Oropharynx	IVa	yes
28	x	x	Oropharynx	IVa	no
29	x	x	oral cavity	IVa	yes
31	x	x	Oropharynx	IVa	yes
33	x	x	Oropharynx	IVa	yes
34	x	x	Larynx	IVa	yes
48	x	x	Nasopharynx	II	yes
49	x	x	Hypopharynx	IVa	yes
51	x	x	Hypopharynx	IVb	yes
52	x	x	Larynx	IVa	yes
53	x	x	oral cavity	III	yes
62	x	x	Oropharynx	IVa	yes
64	x	x	Hypopharynx	IVa	yes
65	x	x	Hypopharynx	IVa	yes
66	x	x	Oropharynx	IVa	yes
67	x	x	Oropharynx	IVa	yes
68	x	x	Oral cavity	I	yes
70	x	x	Hypopharynx	IVa	yes
71	x	x	Larynx	IVa	no
72	x	x	Oropharynx	IVa	yes
73	x	x	Oropharynx	II	yes
74	x	x	Maxillary antrum	III	yes
75	x	x	Oropharynx	IVa	no
77	x	x	Larynx	IVb	no
78	x	x	Oropharynx	III	yes
